# ICLAC Has Released Version 14 of Its Misidentified Cell Line Register: Updating the Global Watchlist of Misidentified Cell Lines

**DOI:** 10.3390/cells15070576

**Published:** 2026-03-24

**Authors:** Ralf Weiskirchen

**Affiliations:** Institute of Molecular Pathobiochemistry, Experimental Gene Therapy and Clinical Chemistry (IFMPEGKC), RWTH University Hospital Aachen, D-52074 Aachen, Germany; rweiskirchen@ukaachen.de

Cell line misidentification, often caused by cross-contamination, mislabeling, or other mix-ups, directly undermines the validity and reproducibility of experimental findings [1,2,3]. The latest and recently launched version of the ICLAC Register (version 14) documents 608 misidentified cell lines, with 560 having no known authentic stock [4]. The register records 163 different contaminants, with HeLa cells accounting for 145 misidentified entries, followed by T-24 and M14 cells as frequent contaminants. These errors cross species boundaries, with 73 lines recognized as interspecies contaminations, leading researchers to work on the wrong tissue or species and draw incorrect conclusions regarding human disease. This results in wasted resources, misleading information in the literature, and potential misdirection of translational and clinical efforts built on flawed models.

The International Cell Line Authentication Committee (ICLAC) was established to address the pervasive issue of false and misidentified cell lines by increasing visibility, promoting awareness, and implementing routine authentication testing as key strategies. The ICLAC mainly focuses on curating the ICLAC Register of Misidentified Cell Lines, systematically listing cross-contaminated or misidentified cell lines based on published data and contributor reports. The register was initially developed by Amanda Capes-Davis and her team from CellBank Australia at the Children’s Medical Research Institute in Westmead, Australia. First published in 2010 as the groundbreaking paper titled “Check your cultures! A list of cross-contaminated or misidentified cell lines,” it laid the foundation for the ICLAC’s ongoing work [5]. Since then, the ICLAC has maintained and expanded the register as a living resource, encouraging researchers and cell banks to submit new information on misidentified lines, authentic stocks, and naming conflicts. This transformation has turned a one-time publication into a community-driven, continuously updated standard.

The release of version 14 of the ICLAC Register of Misidentified Cell Lines on 15 February 2026 signifies a crucial moment for quality control in cell-based research [5]. This edition now documents 608 misidentified cell lines, clearly distinguishing between those with no authentic stock, those with available authentic stocks, and cases that have been re-evaluated and withdrawn as not misidentified. By including authentication guidance, Cellosaurus identifiers, and direct links to source publications and cell banks, the updated register transforms scattered technical knowledge into a practical decision tool for laboratories to consult before working with any cell line.

The ICLAC Register is divided into three main sheet tabs based on the strength and implications of the evidence (Table S1). Sheet tab 1 includes cell lines where no authentic stock is known to exist, meaning the contaminating line has replaced the original cells, and these models should not be used as representations of their claimed donor or tissue. Sheet tab 2 lists cell lines with misidentified stocks, but authentic material is still available from the originator or specific cell banks. The register includes an additional column for these entries, directing users to possible locations and catalog numbers for verified stocks. Sheet tab 3 contains cell lines initially thought to be misidentified but have been withdrawn after re-evaluation of the original data and provenance. This highlights that the register is a curated source of scientific judgment that can change with new evidence. Entries are added after reviewing of cell line provenance and authentication data from multiple sources, published reports, contributor information, cell bank websites, and resources like Cellosaurus [6,7]. They are listed alphabetically, documenting where misidentification was reported without assigning responsibility to specific authors or institutions.

To prevent cell misidentification, researchers and clinicians should prioritize cell line identity as a core quality parameter and follow essential steps. Before beginning any project or introducing a “new” line into the lab, investigators should check the ICLAC Register to verify whether the line is known to be misidentified, whether authentic stocks exist, and where to obtain them. They should conduct authentication testing, for example, by short tandem repeat (STR) profiling, complemented where appropriate by species-level assays such as DNA barcoding or isoenzyme analysis upon receipt and at regular intervals. Results should be compared against trusted reference profiles from cell banks or databases like Cellosaurus [6,7]. Cell lines should be sourced from reputable repositories that conduct routine identity checks and clearly identify problematic lines, while in-house stocks should be maintained under standard operation procedures (SOPs) that minimize cross-contamination.

Beyond individual laboratories, the impact of the new register will depend on how well it is integrated into the broader research ecosystem. Journals can require authors to verify their cell lines against the ICLAC Register and to provide recent authentication data and persistent identifiers such as RRIDs [8,9] and Cellosaurus [6,7] accessions for all immortalized lines used. This builds on evidence showing that formal identifiers are associated with fewer incidences of problematic cell lines in the literature. Funding agencies and institutions can enforce authentication plans and periodic verification as part of grant and core-facility quality standards. Repositories can also play a crucial role by conducting regular identity checks, identifying problematic lines in their collections, and aligning their holdings with ICLAC designations. Coordinated policies at these levels will help ensure that consulting the register and conducting robust authentication become standard practices rather than optional strategies.

Additionally, authentication data and detailed provenance (source, passage, and repository catalog numbers) should be reported in manuscripts and shared with the ICLAC or relevant cell banks when discrepancies are found. This will help to correct issues at a community level rather than within individual laboratories. The Register of Misidentified Cell Lines is licensed under the Creative Commons Attribution-NonCommercial-NoDerivatives 4.0 International [10,11]. This allows journals, funding bodies, and other organizations to link this dataset to their homepages and to share the material in any medium or format.

It is also important to recognize the limitations of the current register. It necessarily reflects cell lines and misidentifications that have come to light through testing and publication, and it will never be fully complete or entirely up to date in fast-moving areas where new lines are frequently established and exchanged. The evidence base is also heterogeneous: some entries are supported by extensive STR profiling, cytogenetics, and tissue comparison, while others rest on more limited data that may warrant further investigation as additional samples become available. For these reasons, the register should be viewed as a critical first checkpoint rather than as an exhaustive guarantee of authenticity, and its continued improvement will depend on active community contributions of authentication data, early-passage material, and careful documentation of cell line provenance.

## Data Availability

No new data were created or analyzed in this study. Data sharing is not applicable to this article.

## Supplementary Materials

The following supporting information can be downloaded at: https://www.mdpi.com/article/10.3390/cells15070576/s1.

## Abbreviations

The following abbreviations are used in this manuscript:

ICLAC	International Cell Line Authentication Committee
RRID	Research Resource Identifier
SOP(s)	Standard operating procedure(s)
STR	Short tandem repeat
